# Combined effect of hydrogen sulfide and mesenchymal stem cells on mitigating liver fibrosis induced by bile duct ligation: Role of anti-inflammatory, anti-oxidant, anti-apoptotic, and anti-fibrotic biomarkers

**DOI:** 10.22038/IJBMS.2021.56477.12604

**Published:** 2021-12

**Authors:** Rehab Ahmed Mohammed, Heba Mohamed Shawky, Laila Ahmed Rashed, Hala Mohamed Elhanbuli, Dalia Nabil Abdelhafez, Eman Sayed Said, Ramadan Mostafa Shamardan, Rania Hosny Mahmoud

**Affiliations:** 1 Department of Physiology, Faculty of Medicine, Fayoum University, Egypt; 2 Department of Physiology, Faculty of Medicine, Cairo University, Egypt; 3 Department of Medical Biochemistry and Molecular Biology, Faculty of Medicine, Cairo University, Egypt; 4 Departments of Pathology, Faculty of Medicine, Fayoum University, Egypt; 5 Departments of Pharmacology, Faculty of Medicine, Fayoum University, Egypt; 6 Department of Pharmacology and Toxicology, College of Pharmacy, Qassim University, Buraydah 52571, Saudi Arabia; 7 Departments of Anatomy, Faculty of Medicine, Fayoum University, Egypt; 8 Departments of Medical Biochemistry and Molecular Biology, Faculty of Medicine, Fayoum University, Egypt

**Keywords:** Bile duct ligation, Hydrogen sulfide, Liver fibrosis, Mesenchymal stem cells

## Abstract

**Objective(s):**

Liver fibrosis eventually develops into cirrhosis and hepatic failure, which can only be treated with liver transplantation. We aimed to assess the potential role of hydrogen sulfide (H_2_S) alone and combined with bone marrow-derived mesenchymal stem cells (BM-MSCs) on hepatic fibrosis induced by bile-duct ligation (BDL) and to compare their effects to silymarin.

**Materials and Methods:**

Alanine aminotransferase (ALT), aspartate aminotransferase (AST), total bilirubin (TB), and alkaline phosphatase (ALP) were investigated in serum. Gene expression levels of CBS (cystathionine β-synthase), CSE (cystathionine γ-lyase), and alpha-smooth muscle actin (α- SMA) were measured in liver tissues using RT-PCR. Hepatic protein kinase (Akt) was assessed by Western blot assay. Liver oxidative stress markers, malondialdehyde (MDA), and reduced glutathione (GSH) were analyzed by the colorimetric method. Lipocalin-2 (LCN2) and transforming growth factor-β (TGF-β) were measured using ELIZA. Liver tissues were examined by H&E and Masson trichome staining for detection of liver necrosis or fibrosis. Caspase 3 expression was evaluated by immunohistochemistry.

**Results:**

H_2_S and BM-MSCs ameliorated liver function and inhibited inflammation and oxidative stress detected by significantly decreased serum ALT, AST, ALP, TB, and hepatic MDA, Akt, TGF-β, LCN2, and α-SMA expression and significantly increased CBS and CSE gene expression levels. They attenuated hepatic apoptosis evidenced by decreased hepatic caspase expression.

**Conclusion:**

Combined treatment with H_2_S and BM-MSCs could attenuate liver fibrosis induced by BDL through mechanisms such as anti-inflammation, anti-oxidation, anti-apoptosis, anti-fibrosis, and regenerative properties indicating that using H_2_S and MSCs may represent a promising approach for management of cholestatic liver fibrosis.

## Introduction

The liver is the largest metabolic organ in the body. It is involved in erythropoiesis, plasma protein synthesis, certain substances detoxification, and bile production and secretion (1). Due to the lack of effective treatment of progressive liver fibrosis so far, it is considered a serious health problem, which eventually develops to cirrhosis and hepatic cell failure with the need for liver transplantation (2). Fibrosis of the liver is a type of wound-healing resulting from liver damage induced by various causes such as autoimmune, infective (mostly hepatitis B and C viruses), metabolic, and drug-induced or toxic causes (3). Liver fibrosis is described by excessive production of extracellular matrix (ECM) that is produced from complex interactions of matrix-forming hepatic stellate cells (HSCs), infiltrating blood, and inflammatory cells (4). HSCs are recognized as the main source of ECM in fibrotic liver (5). They are differentiated into active myofibroblasts and express alpha-smooth muscle actin (α- SMA) due to their activation (6). 

Damage of the hepatocytes is induced by reactive oxygen species (ROS), resulting in the release of mediators that subsequently augment the inflammatory response through production of inflammatory cytokines and growth factors (7). The produced growth factors could induce anti-apoptotic hepatic protein kinase (Akt) phosphorylation and activation (8). It was demonstrated that phosphorylated Akt has an important role in the process of HSCs activation, cell proliferation, and collagen synthesis promoting the accumulation of ECM and progression of hepatic fibrosis. Furthermore, activated signaling molecules like phosphatidylinositol-3-kinase (PI3K) and phosphorylated Akt could affect the apoptotic gene expression levels such as Bax, Bcl-2, and caspase 3 (9).

Silymarin, an extract of *Silybum marianum, *has lipophilic properties and is formed of three flavonolignane diastereomers and two flavonoids. Silymarin has been utilized either alone or as a major ingredient of many pharmacological medications for a long time as a hepatoprotective agent. It has achieved protective effects against inflammation, oxidation, and apoptosis (10). It has been assessed in multiple animal models and performed prominent therapeutic benefits on hepatic damage of various causes, involving alcoholic-induced liver injury and carbon tetrachloride-induced oxidative damage (11).

Hydrogen sulfide (H_2_S) plays crucial regulatory roles in various physiological and pathological conditions. The deficiency of H_2_S is associated with the pathogenesis of fibrosis, causing structural and functional damage of different organs (12). H_2_S is internally generated in different tissues mainly through the transsulfuration pathway by two enzymes, cystathionine β-synthase (CBS) and cystathionine γ-lyase (CSE), with L-cysteine and homocysteine substrates (13).

Decreased production of H_2_S induces increased resistance in cirrhotic livers. The multiple actions of H_2_S include mainly inhibiting inflammatory markers, oxidative stress, lipid peroxidation, increasing the expression of phosphorylated Akt, and activating ATP-dependent potassium (K_ATP_) channels. Furthermore, H_2_S displays vasodilatation and cytoprotective activities. So, it has a possible role in the management of liver fibrosis (14).

Mesenchymal stem cells (MSCs) are adherent, fibroblast-like and pluripotent cells. They have been found to be located in most tissues studied till now, involving adipose tissue, bone marrow, muscular tissue, hair follicles, dermis, and placenta. Studies have elucidated that MSCs could be differentiated into adipogenic, osteogenic, chondrogenic, myogenic, and hematopoietic cells. Moreover, MSCs play an essential role in regeneration of different organs (15). Bone marrow-derived MSCs (BM-MSCs) have confirmed potential therapeutic effects for hepatic fibrosis in various clinical and experimental studies. MSCs have also revealed anti-fibrotic and regenerative effects of damaged liver in experimental liver fibrosis (16).

Bile duct ligation (BDL) is used as a well-known experimental model to develop liver fibrosis in rats. BDL leads to bile flow interruption (cholestasis), which is followed by pathological changes similar to those noticed in biliary cirrhosis. The retained bile acids in liver parenchyma contribute to the damage of hepatocytes and the induced inflammatory reactions (17). Bile duct obstruction in congenital biliary atresia leads to retention of bile and progressive liver fibrosis (18). We aimed to detect for the first time based on our knowledge, the potential therapeutic role of each of H2S and BM-MSCs and the association of each other on liver fibrosis induced by bile-duct ligation in rats and to compare their effects to those of high doses of silymarin which was used as a traditional hepatoprotective drug for decades. 

## Materials and Methods

***Animals***

Forty-two male Albino rats weighing between 180 and 200 gm were used in this study. Rats were purchased and retained in the animal house of the Faculty of Medicine, Cairo University, Cairo, Egypt. Rats were kept in an environment of average temperature of 26 ± 1 °C and humidity (55 ± 5) in repeated cycles of light and dark for an average of 12 hr each. Rats were allowed free access to drinking water and a well-balanced diet. The experimental procedures were documented by the Animal Care Ethical Committee of Faculty of Medicine, Cairo University, Cairo, Egypt. 

***Experimental design***

Induction of liver fibrosis was achieved by common bile duct (CBD) ligation. Surgical procedures were performed as demonstrated.

***Surgical procedures***

All procedures were done under clean conditions. All used instruments were sterilized and animals were kept on a warming plate at 37 °C. BDL operation was done under the effect of diazepam (3 mg/kg) and ketamine hydrochloride (50 mg/kg) anesthesia (19). 

The abdomens of the rats were opened with a midline laparotomy of a length of about 2 cm. The liver was lifted so that the ventral side of it was clearly visible. CBD ligation was carefully separated from the portal vein and hepatic artery. Double ligation at CBD was done without cutting to avoid peritonitis. The abdominal layers were closed with separate sutures and the operation area was sterilized with antiseptic solution (20). Laparotomy was done on sham-operated rats and their common bile ducts were exposed but without ligation. 

The rats were allowed to recover in a cage until they were fully awake and active. Analgesic therapy was given for 1 day. Animals were kept with free access to water and food until the termination of the study. 

***Isolation, preparation, and identification of BM-MSCs***

The bone marrow cells were isolated as explained by Elawady *et al*. (21). Initially, the bone marrow of the tibias and femurs of rats was flushed with phosphate buffer saline (PBS). Over 15 ml of Ficoll-Paque (Gibco-Invitrogen, Grand Island, USA), the flushed bone marrow cells were layered. Following centrifugation, the upper layer was thrown away leaving a layer of nucleated cells which was gathered, washed twice in PBS, and centrifuged. The isolated BM-MSCs were cultured with the addition of 0.5% penicillin/streptomycin and 10% fetal bovine serum (FBS) and incubated at 37 °C and 5% CO_2_ until reaching 80–90% confluence. After 7 days, the cultured BM-MSCs were collected with 0.25% trypsin-EDTA (Gibco, BRL, USA) and resuspended in other flasks. The first passage of cultured BM-MSCs was used in the experiment. The BM-MSCs were identified by their spindle-shaped morphology. Characterization of BM-MSCs was indicated using flow cytometry. BM-MSCs were suspended (1x 10^6^ cells/ml) and incubated with FITC conjugated monoclonal antibodies for CD34, CD105, and CD90 (Biolegend). 

***Labeling of BM-MSCs with PKH26***

The cultured BM-MSCs were identified using PKH26 (Sigma-Aldrich, Saint Louis, MO, USA). PKH26 is a red fluorochrome that binds irreversibly to cell membranes. Cells were first centrifuged, washed twice, and pelleted and suspended in a dye solution. Cells were then injected intravenously into rat tail vain. Liver tissues were investigated after four weeks using a fluorescence microscope to identify and trace the stem cells (22).

***Drugs***

Silymarin (Legalon®140 mg) was introduced from Chemical Industries Development (CID; Giza, Egypt) under license of MADAUS, Germany. Capsules were evacuated then dissolved in distilled water. The solution was freshly prepared. 

***Experimental groups***

Four weeks following BDL or sham operation, measurement of serum bilirubin was done to ensure the success of the operation. Additionally, to the sham group (n=7), BDL rats were further subdivided into 5 groups (7 each). **Group 1**: sham group, 4 weeks after the sham operation, rats were injected with 0.2 ml phosphate-buffered saline (PBS). **Group 2**: control surgical group (BDL group), rats were injected with 0.2 ml PBS. **Group 3**: silymarin group, rats were orally given a high dose of silymarin (200 mg/ kg) daily for four successive weeks (23). **Group 4**: rats were injected intraperitoneally with 1 ml NaHS solution (10 mmol/kg body weight) every 2 days for 4 weeks (24). **Group 5**: BM-MSCs group, rats were injected with a single dose of 0.2 ml PBS containing 3×10^6^ BM-MSCs per rat through tail vein (25). **Group 6**: rats were given an intraperitoneal injection of 1 ml NaHS every 2 days for 4 weeks and 0.2 ml PBS containing 3×10^6^ BM-MSCs as a single dose per rat. 

At the end of treatment, the collected blood was centrifuged (3000g, 4 °C, 20 min). The serum was separated for the analysis of liver function tests. All rats were sacrificed, and hepatic tissues were gathered and divided into two parts; one part for assessment of biochemical markers and the other part for histopathological and immunohistochemical investigations. 

***Measurement of serum and hepatic biochemical parameters***

Serum levels of ALT, AST, total bilirubin (TB), and alkaline phosphatase (ALP) were analyzed using Spinreact diagnostic kits (Spinreact, Spain). For measurement of hepatic malondialdehyde (MDA) and reduced glutathione (GSH), liver tissues were homogenized in ice-cold PBS and these parameters were then investigated by colorimetric method using Biodiagnostic kits (Biodiagnostics, Giza, Egypt). Hepatic lipocalin 2 (LCN2) and transforming growth factor- β (TGF- β) were assayed with ELISA kits (Sunglong Biotech, Hangzhou, China) for rats according to the manufacturers’ instructions.

***Measurement of Akt protein level by Western blot analysis***

Samples of liver tissue were homogenized and lysed on ice with radioimmunoprecipitation assay lysis (RIPA) buffer (Roche, Branchburg, NJ, USA). Total liver protein extracts were separated by 12% sodium dodecyl sulfate-polyacrylamide gel electrophoresis (SDS-PAGE). Proteins were transported electrophoretically to nitrocellulose membranes (Bio-Rad, Hercules, USA). The membranes were blocked with Tris-buffered saline (0.1% Tween 20, 1.5 mM Tris base, 5 mM NaCl, pH 8) containing 5% nonfat dry milk for one hour and incubated with rat anti-Akt (1:1000, ab8805) monoclonal antibody overnight at 4 °C. Proper HRP-conjugated secondary antibody (Rabbit anti-rat IgG-HRP, ab6734) was incubated at room temperature for two hours in 5% nonfat milk dissolved in Tris-buffered saline (TBS) against the blotted target protein. Immunoblots were performed to quantify the levels of Akt protein against the internal control, b-actin (ab227387) by image analysis software produced by Bio-Rad.

***Real-time quantitative polymerase chain reaction “RT-qPCR”***

Total RNA was extracted from homogenates of liver tissue samples using RNeasy Mini Kit (Qiagen, Valencia, USA) according to the manufacturer’s instructions. Reverse transcription reactions were achieved using 2 μg of total RNA and QuantiTect® Reverse Transcription Kit as demonstrated in the manufacturer’s protocol for the production of cDNA (Qiagen, Hilden, Germany). Real-time RT-PCR reactions were performed using gene expression assays for cystathionine β-synthase (CBS), cystathionine γ-lyase (CSE), α- smooth muscle actin (α-SMA), and B-actin (Biotez Berlin-Buch GmbH, Berlin, Germany). The used primer sequences for real-time PCR were as follow: CBS (forward: 5′- CTGTGAAGGGCTATCGCTGC -3′ and reverse: 5′- CTGGCATTGCG GTACTGGTC -3′); CSE (forward: 5′- TGTTGTCATGGGCTTAGTG -3′ and reverse: 5′- CCATCCCATTCCTGAAGTG -3′); α-SMA (forward: ′5- GTGCTGTCCCTCTATGCCTCTGG -3′ and reverse: 5′ -GGCACGTTGTGAGTCACACCATC -3′) and beta-actin (forward: 5′-TCT GGC ACC ACA CCT TCT- ACA ATG-3′ and reverse: 5′- AGC ACA GCC TGG ATA GCA ACG-3′). Quantitative real-time PCR was done in 25 µl reaction volume using 2X SYBR Green PCR Master Mix (Applied Biosystems, Foster City, USA), 2.5 µl of the primer, and 5 µl of cDNA. The following thermal conditions were followed: 95 °C for 15 min as a pre-amplification step, followed by 40 cycles of 94 °C for 15 sec, 60 °C for 30 sec, and 72 °C for 30 sec. Relative expressions of the studied genes were analyzed using the threshold cycle (Ct) method using the equation 2^-ΔΔCt^. The fold-changes (FC) of the expression of studied genes were normalized to the expression levels of the beta-actin gene which was considered as an internal control. Control value was assessed as 1. Values of FC more than 1 indicated up-regulation, while FC values less than 1 revealed down-regulation (26). 

***Histological examination***

Liver samples were kept in 4% buffered paraformaldehyde, embedded in paraffin blocks, and cut into 5-μm thick sections. Tissue slices were stained by Hematoxylin and Eosin (H&E) and Masson trichrome stains and then examined under a light microscope. Assessment of the extent of hepatic fibrosis was performed according to the formula of Scheuer scoring system, with minor modifications. Fibrosis was graded as 0: no fibrosis; grade 1: enlarged, fibrous portal tracts; grade 2: periportal or portal- portal septa, but intact architecture; grade 3: fibrosis with architectural distortion; and grade 4: probable or definite cirrhosis. Furthermore, hepatocyte necrosis or degeneration severity was graded as 0, no hepatocyte necrosis or degeneration; grade 1, focal necrosis or degeneration of hepatocytes (mild, lesion no. ≤ 3); grade 2, multifocal necrosis or degeneration of hepatocytes (moderate, lesion no.> 3); and grade 3, locally extensive or diffuse necrosis or degeneration of hepatocytes (severe) (27). 

***Caspase-3 immunostaining***

For immunohistochemistry using a caspase-3 antibody, deparafﬁnized, rehydrated sections were heated in a microwave oven in citrate buffer (pH 6) for antigen retrieval. The sections were immersed in 0.3% H_2_O_2_ in methanol to block endogenous peroxidase and were pre-incubated with normal mouse serum to block nonspeciﬁc binding of antibodies. After that, the sections were incubated with a primary antibody. The avidin-biotin-peroxidase-complex method was used for detection, with diaminobenzidine tetrachloride as chromogen. The primary antibody used was rabbit polyclonal caspase-3 antibody (CPP32) (1:100; Thermo Fisher Scientific Co., USA). Semiquantitative analysis was performed with scores ranging from ‘0’ for negative staining to ‘1, 2, and 3’ for weak, moderate, and strong cytoplasmic staining, respectively (28). 

***Statistical analysis of data***

The collected data were organized and statistically analyzed using SPSS software statistical computer package version 18 (SPSS Inc, USA). The mean and standard deviation for quantitative data were calculated. ANOVA (analysis of variance) was used to analyze the difference between the mean values of measured parameters among groups. Multiple comparisons between pairs of groups were done using Tukey HSD (*post hoc* range test). Spearman correlation was performed to identify the relations between different study parameters. The significance level was defined at *P*<0.05 for interpretation of the tests’ results.

## Results

***Characterization and hepatic homing of BM-MSCs***

**Figure 1:**
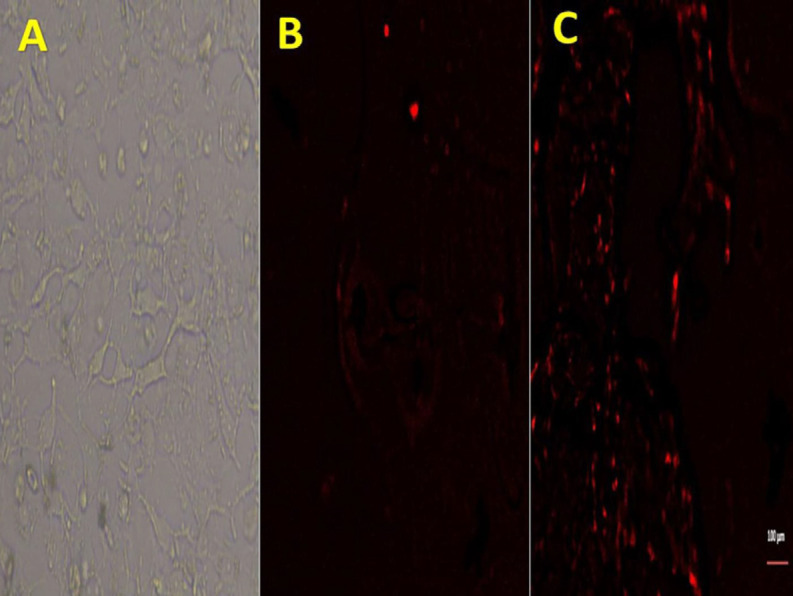
Characterization and hepatic homing of BM-MSCs

**Figure 2:**
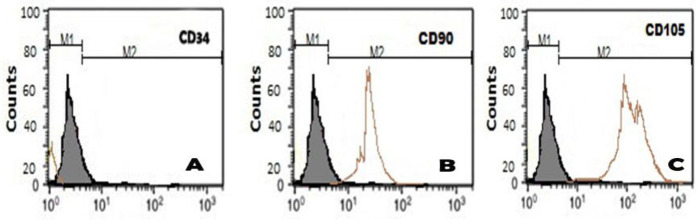
Flow cytometric analysis of BM- MSCs characterization. cells showed positivity for (A) CD34 by 91.8%, (B) CD90 by 85.6%, and (C) CD105 antibodies by 87.2%

Cultured BM-MSCs were identified at 14 days of culture using an inverted microscope by having adherent and fusiform fibroblast-like cells (Figure 1A). By comparison with hepatic tissue of the control group (Figure 1B), BM-MSCs stained with PKH26 fluorescent dye were recognized to be established into the damaged hepatic tissues of rats at 4 weeks after injection by their strong red fluorescence using a fluorescent microscope (Figure 1C). Using flow cytometry analysis, the rat BM-MSCs were positive for CD34 (91.8%), CD90 (85.6%), and CD105 antibodies (87.2%) (Figure 2).

***Effect of H***_2_ ***S and MSCs on liver function, hepatic oxidative stress, and inflammatory markers***

**Figure 3.**
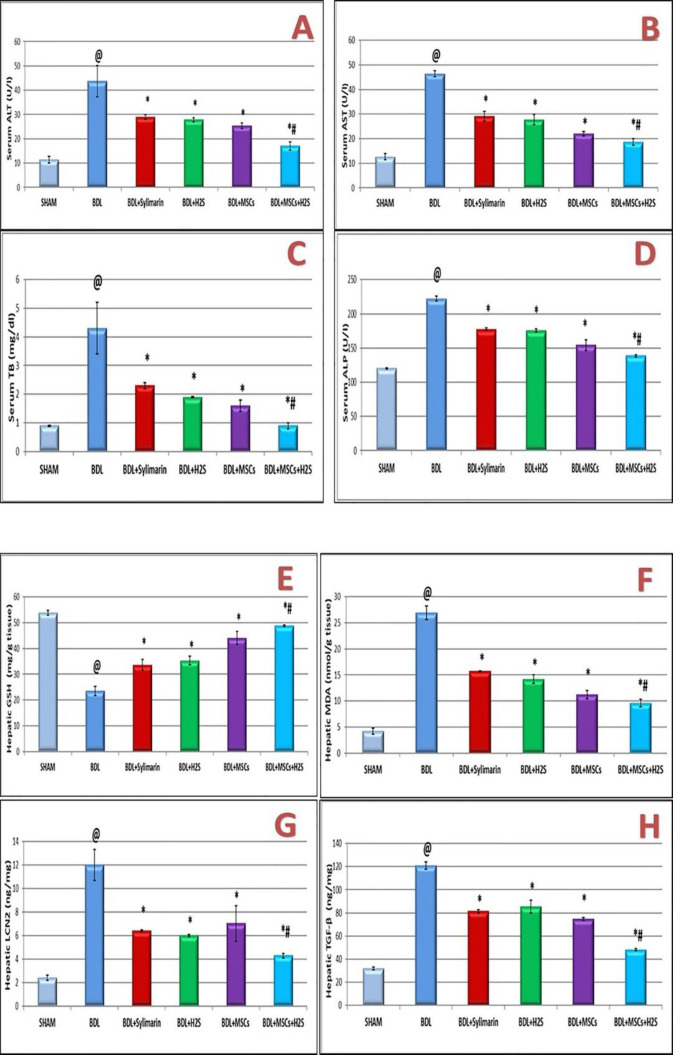
Effect of silymarin, H_2_S, BM-MSCs, and the combined effect of H2S associated with BM-MSCs on liver function, hepatic oxidative stress, and inflammatory biomarkers in BDL rats. The results are expressed as mean ±SD; n= 7 each.* P*@<0.0001 as compared with the sham group, P*<0.0001 as compared with the BDL group, *P*#<0.0001 as compared with the silymarin treated group. BDL, bile duct ligation; BM-MSCs, bone marrow-derived mesenchymal stem cells; H2S, hydrogen sulfide; ALT, Alanine aminotransferase; AST, Aspartate aminotransferase; TB, total bilirubin; ALP, Alkaline Phosphatase; GSH, reduced glutathione; MDA, malondialdehyde; LCN2, lipocalin 2; TGF-β, transforming growth factor-β.* P*<0.05 is considered a significant value

Cholestatic liver injury induced by BDL for four weeks was highly associated with the increase in the levels of serum ALT, AST, total bilirubin (TB), and alkaline phosphatase (ALP), as compared with the sham control group (*P*<0.0001). These changes were concurrent with a significant decrease of hepatic GSH and a marked increase of hepatic MDA level (*P*<0.0001) in BDL-rats compared with the control group (Figure 3). 

Administration of BDL-rats with either silymarin or H_2_S or BM-MSCs significantly decreased ALT, AST, TB, and ALP (*P*<0.0001) compared with the BDL-operated group. The combined effect of H_2_S and BM-MSCs were better than those of silymarin treatment and each of H_2_S and BM-MSCs administration on levels of ALT, AST, TB, and ALP (*P*<0.0001) (Figure 3). A significant increase in serum ALT and AST activities by 282.4% and 269.7%, respectively in BDL-rats when compared with the control group was detected. However, rats administrated with silymarin, H_2_S, or BM-MSCs showed significant decrease in activities of ALT by (34%, 36.3%, and 41.8 %, respectively) and AST by (37.2%, 40.3%, and 52.6%, respectively) compared with the BDL group. The associated treatment of H_2_S and BM-MSCs was coincided with a marked decrease in the activities of ALT by 61.1% and AST by 60% in comparison with the BDL-rats. 

Silymarin, H_2_S, and BM-MSCs treatment is significantly associated with increased hepatic GSH and decreased hepatic MDA (*P*<0.0001) when compared with BDL-operated rats. The combined H_2_S and BM-MSCs treatment significantly elevated hepatic GSH (*P*<0.0001) and lowered hepatic MDA (*P*<0.0001, *P*<0.0001, and *P*=0.001, respectively) as compared with the effect of silymarin and each of H_2_S and BM-MSCs separately (Figure 3). 

There was a significant increase of inflammatory marker, LCN2, and the profibrotic marker, TGF-β in BDL-rats as compared with the sham group (*P*<0.0001). A significant decrease of LCN2 and TGF-β was detected under the effect of silymarin, H_2_S, and BM-MSCs administration when compared with the BDL group of rats (*P*<0.0001). The combined treatment of H_2_S and BM-MSCs decreased LCN2 and TGF-β markedly as compared with the effect of silymarin and each of H_2_S and BM-MSCs separately (*P*<0.001 each) (Figure 3). 

***Effect of H***_2_ ***S and MSCs on cholestatic liver injury***

**Figure 4:**
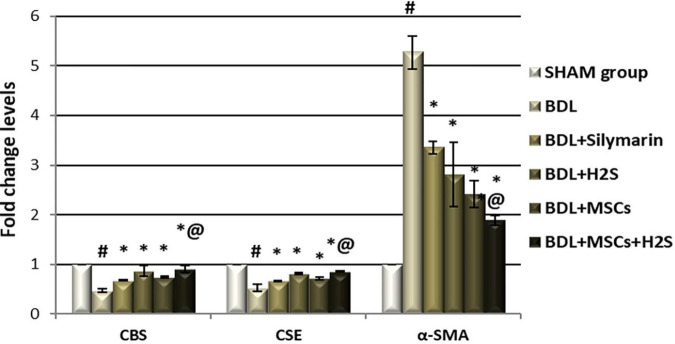
Effect of H_2_S and BM-MSCs administration on gene expression of (A) CBS, (B) CSE, and (C) α-SMA in hepatic tissue of BDL rats. Results were expressed as mean ± SD, n=7 each, threshold cycle (Ct) method was used. The horizontal line represents the expression level of the sham control group. *P*#<0.0001 compared with the sham-operated group, P*<0.0001 compared with the BDL group, and *P*@ <0.0001 compared with the silymarin treated group. The mean value of mRNA expression levels in the control group is defined as 1. The fold change mean levels of more than 1 indicate up-regulation, while values less than 1 represent down-regulation

Cholestatic hepatic injury was associated with significant down-regulation of CBS (0.48 fold change, *P*<0.0001) and CSE (0.53 fold change, *P*<0.0001) gene expression in BDL rats compared with the sham-operated group (Figure 4). BDL-rats treated with silymarin, H_2_S, or BM-MSCs significantly up-regulated the gene expression levels of CBS by (41.25%, 80.21%, and 41.25%, respectively) and CSE by (25.47%, 53.77%, and 36.23%, respectively) compared with the untreated group (*P*<0.0001). Interestingly, the effect of H_2_S treatment was more notable than that stimulated by each of silymarin or BM-MSCs in comparison with BDL-operated rats. 

Moreover, the combined treatment with H_2_S and BM-MSCs significantly increased the expression level of CBS as compared with silymarin (by 33.78 %) and BM-MSCs (by 21.42 %) administrated BDL-rats (*P*<0.0001), while there was an insignificant difference in comparison with H_2_S treated group alone (*P*=0.175). BDL-rats treated with H_2_S and BM-MSCs simultaneously showed significant up-regulation of CSE gene expression when compared with silymarin group and each of H_2_S, as well as BM-MSCs, treated groups separately by 28.87%, 5.15%, and 18.70%, respectively (*P*<0.0001, *P*=0.029, and *P*<0.0001, respectively) (Figure 4).

**Figure 5:**
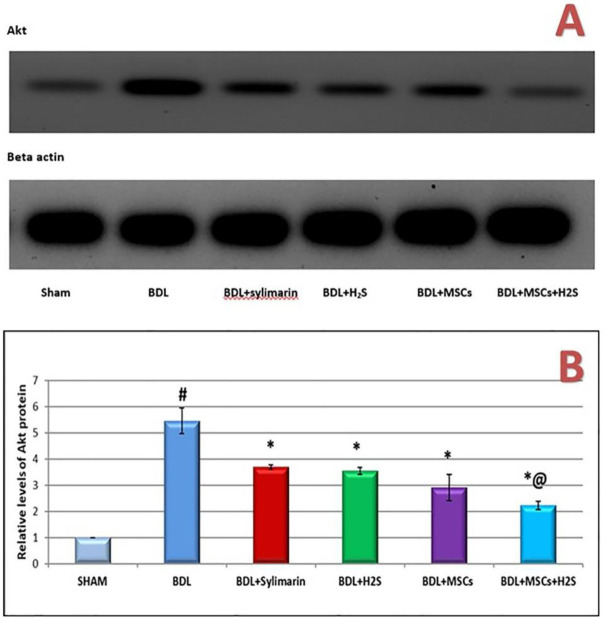
Western blot analysis of Akt protein versus b-actin levels in BDL rats treated with silymarin, H_2_S, MSCs, and combined treatment of H_2_S with MSCs. (A) Representative immunoblots are shown. (B) Mean ratio of Akt against b-actin protein levels in different studied groups. P#, Akt was significantly higher in BDL rats compared with the sham group (*P*<0.0001). P*, silymarin, H_2_S, MSCs, combined MSCs+H2S administration significantly decreased Akt protein expression when compared with the BDL group of rats. *P*@, combined H_2_S and MSCs treatment revealed a significant decrease in the expression level of Akt as compared with the silymarin treated group (*P*<0.0001)

Significant up-regulation in hepatic α-SMA gene expression (5.27 fold change, *P*<0.0001) (Figure 4) and increased Akt protein level measured by Western blot (5.45 folds, *P*<0.0001) (Figure 5) were detected in BDL rats compared with sham-operated group. BDL rats managed with silymarin, H_2_S, or BM-MSCs showed significant down-regulation of α-SMA gene expression levels by 36.43%, 46.68%, and 54.08% when compared with the BDL control group (*P*<0.0001each). Additionally, combined injection of H_2_S and BM-MSCs in BDL rats significantly decreased the expression level of α-SMA by 43.67%, 32.85%, and 22.02% when compared with silymarin, H_2_S and BM-MSCs administrated groups (*P*<0.0001, *P*<0.0001, and *P*=0.003, respectively) (Figure 4). 

Administration of silymarin, H_2_S, and BM-MSCs markedly decreased the level of Akt protein by 32.48%, 35.05%, and 46.7%, respectively (*P*<0.0001) as compared with BDL control rats. However, the effect of BM-MSCs treatment on the level of Akt protein was better than those of each of silymarin and H_2_S (*P*<0.0001) (Figure 5). BDL-rats injected with H_2_S and BM-MSCs simultaneously revealed a significant decrease in Akt protein level (*P*<0.0001) when compared with the silymarin group and each of H_2_S, as well as BM-MSCs treated groups separately by 39.40%, 37.01%, and 23.1%, respectively (Figure 5). Thus, treatment by H_2_S associated with MSCs has an important role in the cholestatic hepatic injury and the developed fibrotic changes management.

***Histological and immunohistochemical examination***

*Hepatic necrosis and fibrosis*

**Figure 6:**
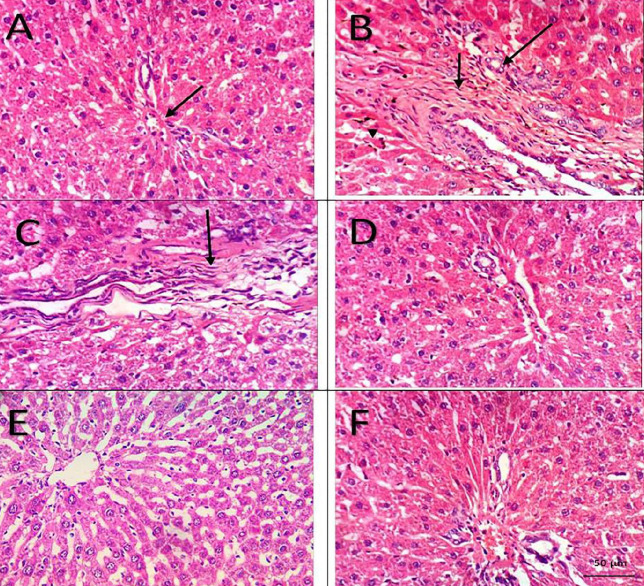
H&E stained sections of hepatic tissue from the 6 studied groups; Sham group: normal portal triad (arrow) (A). BDL group: marked fibroplasia in the portal triad (small arrow) with appearance of newly formed bile ductules (large arrow) and necrosis of sporadic hepatocytes (arrowhead) (B). Silymarin treated group: showing fibroplasia in the portal triad (arrow) (C). H_2_S, MSCs, and H_2_S+MSCs treated groups: no histopathological changes were detected (D, E, F) respectively

Histological examination of H&E stained liver sections (Figure 6) revealed no hepatic necrosis in both of sham group and the group of combined administration of H_2_S and BM-MSCs. All sections from BDL rats were scored as grade 2 hepatic necrosis. Only one rat from BDL rats treated with silymarin showed hepatic necrosis of grade 2 while the rest of the group showed necrosis of grade 1. Only one rat from the group of BDL-rats administrated H_2_S showed hepatic necrosis and it was grade 1. By treatment with BM-MSCs, we detected that only 2 rats had grade 2 hepatic necrosis while the other rats of the group were grade 1. 

**Figure 7:**
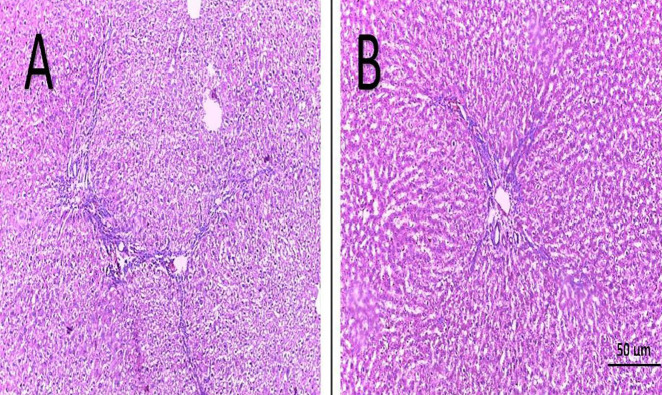
Masson Trichrome stained sections of liver tissues (bluish coloration); all hepatic sections of BDL rats showed bridging fibrosis (A), and reduced fibrous tissue was detected in the silymarin treated group (B). No hepatic fibrosis was found in group 1 (control group), group 4 (which was treated with H_2_S), and all livers from group 6 (combined MSCs+H_2_S treatment) except 2. Hepatic sections with no fibrosis did not take Masson trichrome stain

Evaluation of hepatic fibrosis was done via examination of H&E and Masson Trichrome-stained hepatic sections (Figures 6, 7). No hepatic fibrosis was found in the sham group, BM-MSCs, and combined H_2_S and BM-MSCs treated rats. Furthermore, all livers from H_2_S administrated group showed no hepatic fibrosis except 2 rats were graded as 1. All sections from the BDL group showed hepatic fibrosis of grade 3. Reduced fibrous tissue was detected in the silymarin treated group (Table 1). 

Only the BDL group and BDL group treated with silymarin showed a significant increase in hepatic necrosis and fibrosis when compared with the sham group, while other treated groups showed insignificant differences compared with the sham group (Table 1). There was a significant negative correlation between hepatic fibrosis and necrosis and each of CBS and CSE, and a significant positive correlation between them and Akt, α-SMA, LCN2, and TGF- β (Table 2). 

*Caspase-3 immunostaining*

**Figure 8:**
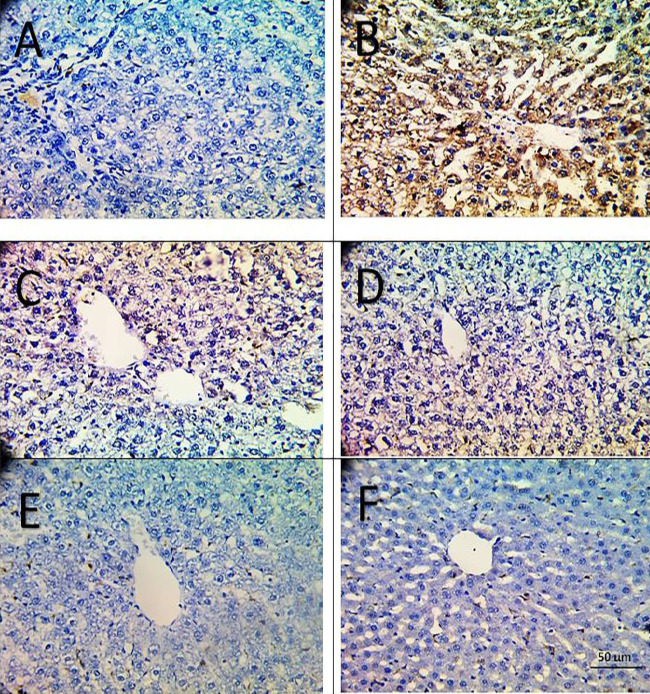
Caspase-3 immunostained sections of hepatic tissue from the 6 studied groups; Sham group: negligible immunopositivity (A). BDL group: increased caspase-3 cytoplasmic expression (B). Silymarin treated group: markedly reduced expression (C). H_2_S, MSCs, and H_2_S+MSCs treated groups: nearly no immunopositivity for caspase-3 (D, E, F), respectively

Immunohistochemical staining for caspase 3 in hepatic tissues (Figure 8) was negligible in the sham, H_2_S, and H_2_S + BM-MSCs groups as it scored zero in all members of these 3 groups. In the BDL group, strong staining was noticed in the livers of only 2 members while the rest of the same group showed a moderate intensity of staining. Three members of the silymarin group showed mild staining while the rest of the same group were negative for caspase-3 immunostaining.

Only BDL and silymarin groups reported a significant increase in caspase-3 immunostaining compared with the sham group, while the other treated groups showed insignificant differences as compared with the sham-operated group (Table 1).

There was a significant negative correlation between caspase immunostaining and each of CBS and CSE, and a significant positive correlation between it and Akt, α-SMA, LCN2, and TGF- β (Table 2). 

Thus, regarding hepatic fibrosis, the group treated with BM-MSCs alone in addition to the group taking combined H2S+ MSCs showed the best results. Moreover, the groups administrated H_2_S alone and H_2_S associated with BM-MSCs revealed the best results regarding the hepatic necrosis score and caspase-3 immunostaining.

(A) Cultured BM-MSCs were identified by having adherent and fusiform fibroblast-like cells. Using fluorescent microscope (Scale bars: 100 μm), BM-MSCs labeled with PKH26 fluorescent dye were demonstrated in (B) hepatic tissue of control group and in (C) damaged liver tissue of BDL rats received 3×106 BM-MSCs per rat through tail vein following 4 weeks of the injection

**Table 1:** The effect of silymarin, H_2_S, BM-MSCs, and combined treatment of H_2_S with BM-MSCs on hepatic necrosis, fibrosis, and Caspase-3 immunostaining in bile-duct ligation (BDL) rats

	Sham	BDL	BDL+ Silymarin	BDL +H_2_S	BDL+ BM-MSCs	BDL+H_2_S + BM-MSCs
Hepatic necrosis	0.0	2± 0.0^a^	0.6± 0.5^*^	0.0^*^^#^	0.14± 0.4^*^^#^	0.0^*^^#^
Hepatic fibrosis	0.0	3± 0.0^a^	0.7± 0.5^*^	0.3± 0.5^*^	0.0^*^^#^	0.0^*^^#^
Caspase-3 immunostaining	0.0	2.3± 0.5^a^	0.4± 0.5^*^	0.0^*^^#^	0.0^*^^#^	0.0^*^^#^

The results are expressed as mean±SD; n= 7 each. *P*a<0.0001 compared with sham operated group, P*<0.0001 compared with BDL rats, *P*#< 0.05 as compared with silymarin treated group. BDL, bile duct ligation; BM-MSCs, bone marrow derived mesenchymal stem cells; H_2_S, hydrogen sulfide. *P*<0.05 is a significant value

**Table 2:** Correlation between hepatic necrosis, hepatic fibrosis, and caspase-3 immunostaining and different parameters in bile-duct ligation (BDL) rats

	CBS R (*P*-value)	CSE r (*P* -value)	AKT r (*P* -value)	α-SMA r (*P* -value)	LCN2 r (*P* -value)	TGF-β r (*P* -value)
H. necrosis	-0.50 (0.001*)	-0.55 (0.000*)	0.67 (0.000*)	0.61 (0.000*)	0.58 (0.000*)	0.65 (0.000*)
H. fibrosis	-0.59 (0.000*)	-0.61 (0.000*)	0.71 (0.000*)	0.72 (0.000*)	0.62 (0.000*)	0.73 (0.000*)
Caspase-3	-0.56 (0.000*)	-0.56 (0.000*)	0.65 (0.000*)	0.63 (0.000*)	0.56 (0.000*)	0.73 (0.000*)

CBS, cystathionine β-synthase; CSE, cystathionine γ-lyase; Akt, hepatic protein kinase; α-SMA, alpha-smooth muscle actin; LCN2, lipocalin-2, TGF-β, transforming growth factor-β. *Significant *P*-value. *P*<0.05 is considered a significant value

## Discussion

Cholestatic liver injury is one of the most important causes of liver fibrosis which leads to cirrhosis and liver cell failure, for which no treatment is available except for liver transplantation. MSCs are known for their regenerative ability and hepatic differentiation potential (29). The protective effects of H_2_S against hepatic fibrosis might be contributed to the inhibition of oxidative stress and inflammation (30). To our knowledge, the present study demonstrates for the first time, the potential therapeutic roles of H_2_S combined with mesenchymal stem cells in cholestatic liver injury produced by ligation of CBD and compares their effects to silymarin as a traditional hepatoprotective drug. 

In the present study, we detected that cholestatic liver injury induced by BDL was associated with significant elevation in serum ALT, AST, TB, and ALP as compared with the sham-operated group. These results were accompanied by a significant reduction in hepatic GSH and a significant increase in hepatic MDA levels.

Our results are consistent with Zheng* et al. *(2013) who determined that ligation of CBD leads to retention of bile in the liver which in turn causes hepatic toxic injury mediated by the retained bile salts which are toxic in high concentrations. Hepatocytes injury was evidenced by increased serum level of hepatic transaminases AST and ALT, as well as increased serum bilirubin and alkaline phosphatase levels in the blood (16). Accumulated bile acids in the hepatocytes are partially responsible for the damage of plasma membranes leading to oxidative stress (31). 

Tahan *et al*. (2010) revealed that oxidative stress is associated with the activation of HSCs, which are the major mediators in the pathogenesis of liver fibrosis. HSCs are activated by MDA, and this activation is inhibited by anti-oxidants (32). GSH is often referred to as the body’s master anti-oxidant, which is an extremely important cell protectant. Increased production of free radicals involving reactive oxygen and nitrogen species leads to parenchymal cell apoptosis and fibroblasts activation (33).

In the current study, we reported significantly elevated levels of TGF-β in the BDL group compared with the control group. Significantly decreased levels of TGF-β in rats administrated with silymarin and each of H_2_S as well as BM-MSCs compared with the BDL group of rats were revealed. Interestingly, combined treatment of H_2_S and MSCs showed a more significant decrease in the level of TGF-β compared with BDL rats. 

Our findings are in agreement with Dooley and ten Dijke (2012) who stated that TGF-β is recognized as a major profibrogenic cytokine included in hepatic fibrosis and its signaling is involved in all disease progression stages, from hepatic injury which include inflammation and fibrosis, to cirrhosis and cancer. High levels of TGF-β, as a consequence of chronic liver damage, are responsible for the transformation of HSCs to myofibroblasts and severe hepatocyte cell death, which leads to the development of liver fibrosis and latterly cirrhosis (34). Additionally, it was found that activated HSCs could produce TGF-β. TGF-β induces transcription of α-SMA and other ECM proteins (35). 

This work showed a significant increase in hepatic LCN2 after cholestatic liver injury, which significantly decreased by administration of combined H_2_S and MSCs simultaneously. In rats, LCN2 is an acute-phase protein that increases quickly in the blood as a result of exposure to toxins or systemic infection. It was revealed that LCN2 levels were related to the degree of acute liver damage and inflammation (36). Many experiments stated that LCN2 overexpression in mice is detected after injection of CCl_4_ (carbon tetrachloride4) and has been associated with liver injury as demonstrated by increased levels of ALT and AST. 

Our results confirm that gene expression of α-SMA is markedly elevated in the BDL group as compared with the control group. Furthermore, treatment of combined H_2_S and MSCs significantly decreases the expression level of α-SMA compared with cholestatic liver injury group. α-SMA expression is a potential biomarker of HSCs activation which contributes to the deposition of fibrous tissue. It was considered to be useful in identifying the primary stages of hepatic fibrosis and following up the efficiency of the therapy (37).

The present study showed that BDL down-regulated the gene expression levels of CBS and CSE. Treatment of H_2_S, as well as H_2_S associated with BM-MSCs, significantly induces the expression of both biomarkers as compared with the group of cholestatic liver injury. NaHS up-regulates hepatic CBS and CSE expression accounting for the production of H_2_S in hepatic tissues. It inhibits cell apoptosis through decreased caspase-3 activity. CSE is the main enzyme responsible for 97% of hepatic production of H_2_S (38).

Furthermore, we demonstrated that ligation of CBD could lead to a marked increase in Akt protein expression as detected by Western blot. H_2_S significantly decreased Akt protein expression when compared with the BDL group of rats. Interestingly, combined H_2_S and MSCs treatment revealed more reduction in the expression levels of Akt compared with BDL rats. Akt played crucial roles in the development of hepatic fibrosis by regulating activation of HSCs and hepatic sinusoid capillarization (39). It was clarified that H_2_S inhibited the expression of phosphorylated Akt. Inhibition of Akt phosphorylation suppresses the activation of HSCs, decreases ECM formation, and stimulates HSCs apoptosis (40). So, the hepatic Akt should be suggested as a potential target in the management of hepatic fibrosis.

We reported that retention of bile acids in the hepatocytes leads to their apoptosis evidenced by increased cytoplasmic caspase 3 measured by immunohistochemistry. Additionally, by histological assessment, it was found that cholestatic liver injury was responsible for the development of hepatic necrosis of grade 2 and fibrosis of grade 3. Simultaneous administration of H_2_S and BM-MSCs revealed no hepatic necrosis and fibrosis. Immunohistochemical staining for caspase 3 in hepatic tissues was negligible in all liver sections of the group of combined H_2_S and BM-MSCs treatment as it scored zero in all members of the group. Thus, we demonstrated that the combined treatment of associated H_2_S with BM-MSCs could give better results than each of them alone and from the silymarin-treated group.

Many studies have revealed that administration of NaHS, a donor of H_2_S, relieves liver injury induced by hepatic ischemia-reperfusion and CCl4-induced acute hepatotoxicity evidenced by histopathological changes and suppression of cell apoptosis (24, 41).

The use of BM-MSCs has been suggested as a new potential therapeutic approach in chronic liver failure. Bone marrow cells are easily available and are becoming an alternative option to liver transplantation (42). The protective effect and the regenerative capacity of MSCs were confirmed by Kisseleva* et al. *(2010) (43).

In this study, silymarin was able to perform favorable effects improving liver function tests in rats of cholestatic liver injury and exerted significant results on total bilirubin and liver enzymes compared with the untreated BDL rats. In cholestatic liver fibrosis, the therapeutic potentials of silymarin may be attributed to its anti-oxidant and anti-fibrotic activity (44). The anti-hepatotoxic mechanism of silymarin is related to its supporting effect on plasma membranes (11). As we detected, silymarin regenerative abilities were less than that of H_2_S and BM-MSCs. Histopathological investigations confirmed the biochemical assessments. 

Our work proved that the use of NaHS alone or in combination with BM-MSCs in a rat model of cholestatic liver injury could attenuate hepatotoxicity, and decrease hepatocytes injury evidenced by decreased ALT, AST, total bilirubin, and serum alkaline phosphatase levels. NaHS associated with MSCs has significantly improved liver fibrosis as well as necrosis, as confirmed by histological examination. This was through their anti-oxidative effect evidenced by increased GSH and decreased MDA levels, and anti-inflammatory effect evidenced by the decreased level of hepatic lipocalin-2. Combined H_2_S and BM-MSCs therapy markedly ameliorated hepatic fibrotic lesions, as evidenced by decreased expression levels of α-SMA, TGF-β, and Akt. Thus, the combined effect of H_2_S and BM-MSCs in liver fibrosis induced by BDL is a better choice than the utilization of each of them alone and the utilization of high doses of silymarin which was used for centuries as a traditional hepatoprotective agent. 

## Conclusion

We suggested, for the first time to our knowledge, that the combined H_2_S and mesenchymal stem cells may act as potential therapeutic agents for liver fibrosis by multiple functions such as anti-oxidation, anti-inflammation, anti-fibrosis, and regenerative properties, indicating that the efficient utilization of H_2_S and MSCs is considered a promising approach for the treatment of cholestatic liver injury.

## Authors’ Contributions

RAM, HMS, and LAR Suggestion of study concept and design; LAR and RHM Interpretation and analysis of biochemical data; HME and DNA Analysis of histopathological results; RAM, RHM, and ESS Writing the article; ESS and RMS Checked the data and revision of the manuscript; all authors final revision and approval of the manuscript. 

## Funding

No funding was received .

## Coflicts of Interest

The authors have no conflicting financial interest.
